# Clinical Significance of Medial Versus Lateral Compartment Patellofemoral Osteoarthritis: Cross‐Sectional Analyses in an Adult Population With Knee Pain

**DOI:** 10.1002/acr.23110

**Published:** 2017-06-27

**Authors:** Vincent Ukachukwu, Rachel Duncan, John Belcher, Michelle Marshall, Joshua Stefanik, Kay Crossley, Martin J. Thomas, George Peat

**Affiliations:** ^1^ Arthritis Research UK Primary Care Centre, Research Institute for Primary Care & Health Sciences, Keele University, Keele, Staffordshire, UK, and Moss Lane Surgery, Moss Lane Madeley Crewe UK; ^2^ Institute of Health & Society, Newcastle University Newcastle upon Tyne UK; ^3^ Arthritis Research UK Primary Care Centre, Research Institute for Primary Care & Health Sciences, Keele University Keele Staffordshire UK; ^4^ Northeastern University Boston Massachusetts; ^5^ La Trobe University Melbourne Australia

## Abstract

**Objective:**

To determine the comparative prevalence, associations with selected patient characteristics, and clinical outcomes of medial and lateral compartment patellofemoral (PF) joint osteoarthritis (OA).

**Methods:**

Information was collected by questionnaires, clinical assessment, and radiographs from 745 eligible community‐dwelling symptomatic adults age ≥50 years. PF joint space narrowing (JSN) and osteophytes were scored from skyline radiographs using the Osteoarthritis Research Society International atlas. Multilevel models were used to assess associations of compartmental PF joint OA with age, sex, body mass index (BMI) and varus–valgus malalignment, while median regression was used to examine associations with clinical outcomes (current pain intensity on a numeric rating scale [0–10] and the function subscale of the Western Ontario and McMaster Universities Osteoarthritis Index [0–68]).

**Results:**

Isolated lateral PF joint OA was more common than isolated medial PF joint OA, particularly at higher severity thresholds. Irrespective of severity threshold, age (≥2 odds ratio [OR] 1.19 [95% confidence interval (95% CI) 1.12, 1.26]), BMI (≥2 OR 1.15 [95% CI 1.07, 1.24]), and valgus malalignment (≥2 OR 2.58 [95% CI 1.09, 6.07]) were associated with increased odds of isolated lateral JSN, but isolated medial JSN was only associated with age (≥2 OR 1.20 [95% CI 1.14, 1.27]). The pattern of association was less clear for PF joint osteophytes. Isolated lateral PF joint OA, defined by JSN or osteophytes, was associated with higher pain scores than isolated medial PF joint OA, but these differences were modest and were not significant. A similar pattern of association was seen for functional limitation but only when PF joint OA was defined by JSN.

**Conclusion:**

Isolated lateral PF joint OA is more common than isolated medial PF joint OA, and it is more consistently associated with established OA risk factors. It is also associated with higher, but clinically nonsignificant, pain and function scores than isolated medial PF joint OA, particularly when PF joint OA is defined using JSN.

## INTRODUCTION

Patellofemoral (PF) joint osteoarthritis (OA) contributes to knee pain and functional limitation 1, 2, 3 and may be a target for the early management of knee OA 4. Effective conservative management options for PF joint OA are lacking, with trials of bracing, taping, and exercise yielding conflicting results 5, 6, 7, 8. These conservative treatment strategies, as well as some surgical treatments, largely attempt to realign the patella medially to unload the lateral PF joint compartment. This is because PF joint OA is believed to be predominantly a disease of the lateral PF joint compartment based on the “law of valgus” put forward by Ficat and Hungerford in their seminal work on the disorders of the PF joint 9. The law suggests that the predominant frontal plane force acting on the patella during knee motion is directed laterally, leading to excessive loading of the lateral facet of the PF joint. This is in line with more recent biomechanical studies of the PF joint that report higher loading of the lateral PF joint facet 10, 11, 12, 13, with the lateral facet contact force estimated to be 4–6 times higher than the medial facet contact force 13.

Box 1 **Significance & Innovations**

- While mild patellofemoral (PF) osteoarthritis (OA) is equally common in both compartments of the PF joint, moderate to severe disease more commonly affects the lateral compartment.
- Isolated lateral PF joint OA is associated with more pain and reduced function than isolated medial PF joint OA, but these differences are modest and clinically nonsignificant. The relationship between compartmental radiographic PF joint OA and clinical outcomes may vary depending on the morphologic feature and severity threshold used to define PF joint OA.
- Our findings support better patient selection for clinical trials, e.g., through the inclusion of symptomatic patients with probable PF joint space narrowing (grade ≥1), and possibly the need to rethink current PF joint OA treatments that attempt to realign the patella medially.

However, a higher prevalence of OA in the lateral PF joint compartment is not consistently found in epidemiologic studies. In one of the earliest studies, of 66 orthopedic clinic patients with relatively severe knee OA, lateral PF joint disease was found on plain radiography in 89% of knees, compared with medial PF joint disease in only 11% of knees 14. Elahi et al included the dominant knees of 292 participants recruited from the community and showed that 67 of 86 participants with PF joint OA had evidence of predominantly lateral PF joint OA, whereas 19 had evidence of predominantly medial PF joint OA 15. In contrast, the higher frequency of lateral PF joint OA observed in these studies was recently challenged by evidence from large‐scale magnetic resonance imaging (MRI) studies of knee OA, which reported PF cartilage damage and bone marrow lesions (BMLs) in the medial compartment at least as often as in the lateral 16, 17, 18, 19 (see Supplementary Table 1, available on the *Arthritis Care & Research* web site at http://onlinelibrary.wiley.com/doi/10.1002/acr.23110/abstract).

However, the clinical significance of the MRI lesions in the medial PF joint compartment is unclear. Stefanik et al observed more common and more severe knee pain when OA involved the lateral PF joint compartment, despite the high prevalence of MRI‐detected cartilage damage in the medial PF joint compartment 20.

Studies of risk factors for compartmental PF joint OA have shown an association with patellar dislocation or subluxation 14, patellar malalignment 21, 22, 23, and varus–valgus malalignment 15, 24. The established OA risk factors of age, sex, and body mass index (BMI) have also been studied in relation to the PF joint 1, 25, but the association of these risk factors with compartmental PF joint OA has not been specifically assessed. Furthermore, the association of PF joint OA with pain and functional limitation is now well‐recognized, but there is limited evidence on the relative contribution of compartmental PF joint disease to these outcomes 20. Understanding the relative prevalence, association with risk factors, and clinical significance of medial and lateral compartment PF joint OA may lead to a better understanding of the etiology of PF joint OA and inform the development of treatment strategies as well as the design of clinical trials. Using cross‐sectional baseline data from the Clinical Assessment Study of the Knee (CAS‐K), a cohort of community‐dwelling adults (age ≥50 years) with knee pain, our aim was to determine the relative frequency, the direction and magnitude of the association with selected risk factors, and the strength of the association with pain and functional limitations in lateral versus medial PF joint OA. We hypothesized that the findings depended on the morphologic feature and severity threshold chosen and that this, therefore, might be one of the reasons for the conflicting findings reported in previous studies.

## PATIENTS AND METHODS

#### Study population

The CAS‐K is a prospective, population‐based, observational cohort study of knee pain in adults age ≥50 years. Individuals reporting knee pain in the past year were identified through a mailed survey and invited to attend a research clinic, where a detailed clinical assessment was performed and bilateral knee radiographs were taken. The CAS‐K cohort design, methods, and recruitment have been described in detail elsewhere 26, 27. Ethical approval was obtained for all stages of the study, and participants gave written informed consent.

#### Data collection

##### Definition of medial and lateral PF joint OA using plain radiography

Three views of the knee were obtained: a weight‐bearing posteroanterior (PA) semiflexed 28, skyline, and lateral views. The lateral and skyline views were obtained in a supine position with the knees flexed to 45 degrees. The PA view and the posterior aspect of the lateral view were used to assess the tibiofemoral (TF) joint, while the PF joint was assessed using the skyline view. Six radiographers who had been trained to standardize the radiographs performed all of the imaging in a single radiology department, and regular quality control sessions were held. A single reader (RD), blinded to clinical and questionnaire data, scored all of the study radiographs at baseline. Individual radiographic features on the PA and skyline views were scored using the Osteoarthritis Research Society International (OARSI) atlas 29. Osteophytes on the posterior tibial surface do not appear in the atlas but were judged on the same basis of severity as other osteophytes in the lateral view. There was good intraobserver and interobserver repeatability (κ = 0.46–0.86) for scores of individual radiographic features in the skyline views using the OARSI atlas. Further details on the radiographic scoring and definitions have been published previously 30.

For each radiographic feature (joint space narrowing [JSN] and osteophytes), we classified the PF joint (irrespective of TF joint involvement) into 1 of 4 mutually exclusive categories based on the pattern of compartmental involvement: isolated medial, isolated lateral, mixed medial and lateral, and neither medial nor lateral (neither). A sliding threshold definition of compartmental PF joint OA was applied based on the severity of individual radiographic features at grade ≥1 (mild), ≥2 (moderate) and ≥3 (severe). The radiographic severity of TF joint OA was classified as none, mild, moderate, or severe using a combined scoring system (described previously) 31.

##### Risk factors

Age, sex, and BMI were recorded at baseline. Frontal plane knee malalignment was assessed using the proxy clinical measurements of intercondylar and intermalleolar distances performed on standing. Varus and valgus malalignment were defined as intercondylar and intermalleolar distances >0 cm, respectively.

##### Clinical outcomes

Knee pain severity and self‐reported functional limitation were studied. Data on pain were collected in the clinic, using the Chronic Pain Grade scale 32, which included an 11‐point numeric rating scale (NRS; range 0–10) for current knee pain intensity. Participants were asked to score the severity of pain in the index knee, which was identified by the participant as the more problematic knee or chosen at random when both knees were equally symptomatic. Patient‐reported functional limitation was assessed using Western Ontario and McMaster Universities Osteoarthritis Index function subscale scores (range 0–68) 33.

#### Statistical analysis

First, we calculated the prevalence of compartmental PF joint OA by radiographic feature using the sliding cutoff for severity threshold as described above. Subsequent analyses of association with selected patient characteristics and clinical outcomes were limited to thresholds grades ≥1 and ≥2, as there were insufficient numbers in the most severe category (JSN/osteophyte grade 3) to run regression models. We then used knee‐level (1,475 knees) multilevel multinomial regression models taking into account the clustering of both knees in the same subject to determine the associations between selected patient characteristics (age, sex, BMI, and frontal plane knee malalignment) and compartmental PF joint OA. Finally, quantile (median) regression models (performed at the person level, with 745 index knees) were used to assess the association between compartmental PF joint OA and the clinical outcomes of pain and functional limitation, adjusting for age, sex, BMI, and severity of TF joint OA. We compared pain and function scores in each compartmental PF joint OA category, and at each threshold, using the “neither” category as a reference.

## RESULTS

Of the 819 people who attended the research clinic for an assessment, 745 were eligible for the current analysis. Of these, 55% were women, the mean ± SD age was 65.2 ± 8.6 years, and the mean ± SD BMI was 29.6 ± 5.2 kg/m^2^. Reasons for ineligibility were the following: no current or recent knee pain 32, participants declined radiography 2, existing diagnosis of inflammatory arthritis, verified by medical records 16, total knee replacement (TKR) of index knee 15, unlabeled PA view 2, absent patella 2, and skyline views considered uninterpretable 5. These 745 individuals contributed 1,475 knees to the analysis (excluding 15 TKRs of nonindex knees).

#### Relative frequency of compartmental radiographic PF joint OA

Using skyline JSN to define PF joint OA, the prevalence of isolated medial PF joint OA was similar to that of isolated lateral PF joint OA at the mild threshold (defined as JSN ≥1), but at the moderate (JSN ≥2) and severe (JSN = 3) thresholds, isolated lateral PF joint OA was more common than isolated medial PF joint OA. When defined using skyline osteophytes, the prevalence of isolated lateral PF joint OA was higher than that of isolated medial PF joint OA across all severity thresholds (Table 1).

**Table 1:** Relative frequency of medial and lateral PF joint involvement from plain radiographs of 1,475 knees (745 persons), by morphologic feature and severity threshold (OARSI atlas)^a^

	**Severity threshold**		
**Morphologic feature**	**≥1**	**≥2**	**≥3**
JSN			
Neither medial nor lateral PF joint JSN	935 (63)	1,184 (80)	1,303 (88)
Isolated medial PF joint JSN	197 (13)	97 (7)	42 (3)
Isolated lateral PF joint JSN	240 (16)	162 (11)	121 (8)
Mixed medial and lateral PF joint JSN	103 (7)	32 (2)	9 (1)
Osteophytes			
Neither medial nor lateral PF joint osteophytes	576 (31)	1,118 (76)	1,299 (88)
Isolated medial PF joint osteophytes	158 (11)	105 (7)	52 (4)
Isolated lateral PF joint osteophytes	286 (19)	162 (11)	101 (7)
Mixed medial and lateral PF joint osteophytes	455 (31)	90 (6)	23 (2)

a Values are the number (%). PF = patellofemoral; OARSI = Osteoarthritis Research Society International; JSN = joint space narrowing.

#### Compartmental radiographic PF joint OA: association with selected patient characteristics

Multilevel multinomial regression analyses showed that, irrespective of the severity threshold chosen, isolated lateral PF joint OA, when defined by JSN, was associated with greater age (≥1 odds ratio [OR] 1.15 [95% confidence interval (95% CI) 1.11, 1.20] and ≥2 OR 1.19 [95% CI 1.12, 1.26]), higher BMI (≥1 OR 1.15 [95% CI 1.09, 1.22] and ≥2 OR 1.15 [95% CI 1.07, 1.24]), and valgus malalignment (≥1 OR 2.12 [95% CI 1.07, 4.18] and ≥2 OR 2.58 [95% CI 1.09, 6.07]), with varus malalignment appearing to be protective (≥1 OR 0.18 [95% CI 0.07, 0.46] and ≥2 OR 0.30 [95% CI 0.09, 0.90]), while isolated medial PF joint OA was only associated with age (≥1 OR 1.12 [95% CI 1.08, 1.17] and ≥2 OR 1.20 [95% CI 1.14, 1.27]). When defined using osteophytes, both isolated lateral PF joint OA and isolated medial PF joint OA were associated with greater age and higher BMI at both thresholds, while isolated medial PF joint OA, but not isolated lateral PF joint OA, was associated with varus malalignment at the moderate threshold (defined as ≥2). Men in this cohort were found to have higher odds of having PF joint osteophytes, but this association was only seen at the mild threshold (defined as ≥1) (Table 2).

**Table 2:** Risk factor associations (multilevel, multinomial regression analysis using data from 1,475 knees from 745 participants)^a^

	**Isolated medial** **OR_**adj**_ (95% CI)**	**Isolated lateral** **OR_**adj**_ (95% CI)**	**Mixed** **OR_**adj**_ (95% CI)**
Joint space narrowing			
≥1 (ref. neither medial nor lateral)			
Age (ref. per year)	1.12 (1.08, 1.17)^b^	1.15 (1.11, 1.20)^b^	1.20 (1.15, 1.25)^b^
Female (ref. male)	1.06 (0.55, 2.02)	0.76 (0.40, 1.43)	1.15 (0.55, 2.39)
BMI (ref. per kg/m^2^)	1.06 (0.99, 1.13)	1.15 (1.09, 1.22)^b^	1.14 (1.07, 1.22)^b^
Varus (ref. neither)	0.71 (0.31, 1.62)	0.18 (0.07, 0.46)^b^	0.90 (0.35, 2.27)
Valgus (ref. neither)	1.61 (0.80, 3.26)	2.12 (1.07, 4.18)^b^	2.18 (1.00, 4.74)
≥2 (ref. neither medial nor lateral)			
Age (ref. per year)	1.20 (1.14, 1.27)^b^	1.19 (1.12, 1.26)^b^	1.23 (1.15, 1.32)^b^
Female (ref. male)	0.97 (0.41, 2.31)	0.76 (0.34, 1.72)	1.23 (0.42, 3.62)
BMI (ref. per kg/m^2^)	1.01 (0.93, 1.10)	1.15 (1.07, 1.24)^b^	1.07 (0.97, 1.19)
Varus (ref. neither)	0.58 (0.19, 1.80)	0.30 (0.09, 0.90)^b^	0.65 (0.16, 2.58)
Valgus (ref. neither)	2.49 (0.99, 6.24)	2.58 (1.09, 6.07)^b^	1.60 (0.50, 5.09)
Osteophytes			
≥1 (ref. neither medial nor lateral)			
Age (ref. per year)	1.13 (1.08, 1.19)^b^	1.15 (1.10, 1.20)^b^	1.18 (1.12, 1.23)^b^
Female (ref. male)	0.31 (0.14, 0.70)^b^	0.21 (0.09, 0.46)^b^	0.25 (0.11, 0.53)^b^
BMI (ref. per kg/m^2^)	1.25 (1.15, 1.36)^b^	1.24 (1.14, 1.34)^b^	1.34 (1.23, 1.45)^b^
Varus (ref. neither)	1.68 (0.63, 4.50)	1.15 (0.44, 3.02)	2.07 (0.81, 5.27)
Valgus (ref. neither)	1.00 (0.42, 2.40)	2.25 (0.99, 5.11)	1.43 (0.64, 3.22)
≥2 (ref. neither medial nor lateral)			
Age (ref. per year)	1.22 (1.11, 1.35)^b^	1.20 (1.09, 1.33)^b^	1.22 (1.10, 1.35)^b^
Female (ref. male)	1.27 (0.50, 3.19)	1.44 (0.60, 3.47)	1.16 (0.45, 2.97)
BMI (ref. per kg/m^2^)	1.31 (1.14, 1.50)^b^	1.24 (1.08, 1.42)^b^	1.32 (1.15, 1.51)^b^
Varus (ref. neither)	5.02 (1.53, 16.44)^b^	1.87 (0.57, 6.12)	3.69 (1.07, 12.75)^b^
Valgus (ref. neither)	1.10 (0.40, 3.05)	2.25 (0.88, 5.76)	2.40 (0.88, 6.57)

a Odds ratios adjusted for age, sex, BMI, and malalignment, taking into account the clustering of knees within subjects. OR_adj_ = adjusted odds ratio; 95% CI = 95% confidence interval; ref. = reference; BMI = body mass index.

b Statistically significant.

#### Compartmental radiographic PF joint OA: association with clinical outcomes

**Figure 1:**
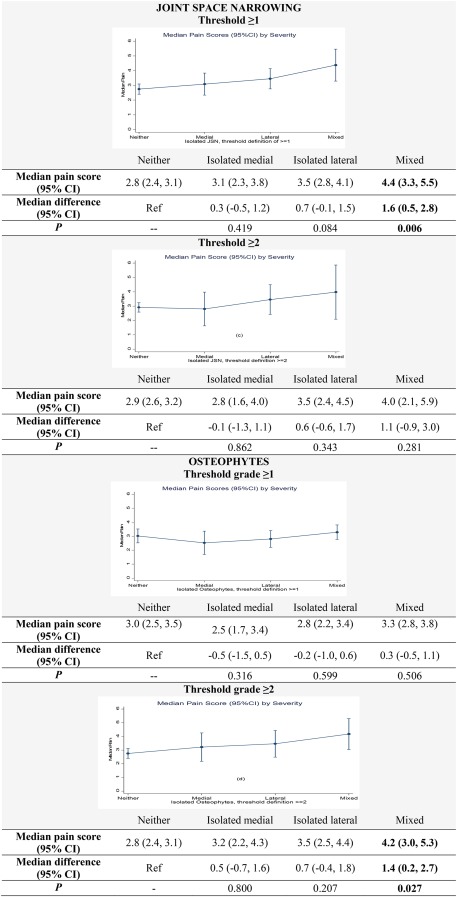
Association of compartmental PF joint OA with pain. 95% CI = 95% confidence interval.

In quantile (median) regression, after adjusting for covariates, median pain scores for isolated lateral PF joint OA were higher than those for isolated medial PF joint OA, whether defined by JSN or osteophytes and irrespective of severity threshold. However, the difference in median pain intensity was generally less than 0.5 points on the 11‐point NRS (Figure 1). When compared with knees with neither medial nor lateral PF joint OA, the difference in median pain intensity was only statistically significant for knees with mixed compartmental PF joint OA and only at the JSN grade ≥1 (*P* = 0.006) and osteophyte grade ≥2 (*P* = 0.027) thresholds (Figure 1).

**Figure 2:**
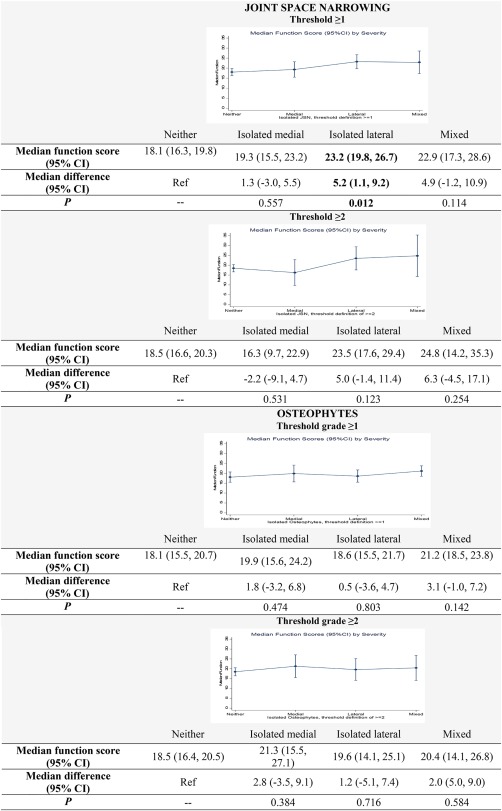
Association of compartmental PF joint OA with functional limitations. 95% CI = 95% confidence interval. Color figure can be viewed in the online issue, which is available at http://onlinelibrary.wiley.com/doi/10.1002/acr.23110/abstract.

In similar regression models but with the WOMAC function subscale score as the outcome, median function scores for isolated lateral PF joint OA were higher than those of isolated medial PF joint OA, but only when defined by JSN (Figure 2). The observed magnitude of difference was roughly 4–7 points on the 0 to 68‐point scale. When compared with knees with neither medial nor lateral PF joint OA, the difference in median function scores was statistically significant for knees with isolated lateral PF joint OA but only at the JSN grade ≥1 (*P* = 0.012) threshold (Figure 2).

## DISCUSSION

Our cross‐sectional study using plain radiography in community‐dwelling symptomatic adults suggests that isolated lateral PF joint OA is more common than isolated medial PF joint OA, particularly at the higher severity thresholds, and is more consistently associated with established OA risk factors. Additionally, we show that the pattern of associations of compartmental PF joint OA with selected risk factors and clinical outcomes differs depending on the morphologic feature and severity threshold used to define PF joint OA, and we suspect that this may be one reason for the conflicting results of clinical trials to date.

The prevalence and risk factor associations of isolated medial and isolated lateral PF joint OA varied depending on the radiographic feature (JSN versus osteophytes) and the severity threshold (mild, moderate, or severe) chosen. Our finding of a higher prevalence of moderate to severe PF joint OA in the lateral compartment than in the medial compartment is consistent with other radiographic studies 14, 15 but at odds with MRI studies, which have found an equal or even greater frequency of medial PF joint cartilage damage and BMLs than lateral 16, 20. Our results, however, further suggest a roughly equal frequency of isolated medial and isolated lateral JSN at the mild JSN (JSN ≥1) threshold. Cahue et al 24 showed that patellofemoral progression was more common in the lateral compartment than in the medial, and it could be that even though early PF joint OA is equally common in both compartments, the faster progression in the lateral compartment means that moderate to severe disease is found more commonly in the lateral PF joint compartment than in the medial. The variance of our results with those of MRI studies may simply reflect differences in the sensitivity of the two imaging modalities, as lateral PF joint cartilage damage and BMLs on MRI were more frequent than those of the medial joint compartment at the most severe thresholds 16.

PF joint OA is associated with the traditional OA risk factors of age, sex, and BMI 1, 25. Our findings show that the association of PF joint OA, whether defined by JSN or osteophytes, with increasing age holds true for both compartments of the PF joint, irrespective of the severity threshold applied. With respect to sex, we unexpectedly found reduced odds of PF joint osteophytes among symptomatic women at the mild threshold (osteophytes ≥1). Other studies have not shown a sex difference in the risk of compartmental PF joint OA 15, and, indeed, previous population‐based studies have found a higher prevalence of radiographic OA in women than in men 34, 35. A male preponderance of symptomatic OA in our cohort has been previously reported 36, 37 and is thought to be due to selective nonparticipation of older symptomatic women and possibly occupational differences between the men and women in our cohort 37.

At both severity thresholds used in this study, higher BMI and valgus malalignment were associated with higher odds of isolated lateral JSN, with varus malalignment appearing to be protective. Isolated medial JSN was only associated with age. The reason for this is not clear. A possible explanation is that different processes drive JSN in the medial and lateral PF joint compartments, with lateral JSN being largely driven by load‐related damage (BMI and malalignment) and medial JSN more to age‐related degeneration, probably driven by a relative lack of loading, which could impair cartilage nutrition 38, 39. However, our finding of an association of isolated medial PF joint osteophytes with load‐related risk factors of BMI and varus malalignment (at the ≥2 threshold) does not support the notion of a lack of loading of the medial PF joint compartment. Furthermore, in contrast with our results, Elahi et al 15 showed that lateral PF joint JSN was associated with valgus malalignment and medial PF joint JSN with varus malalignment but found no association between compartmental PF joint JSN and BMI. The absence of an association between varus malalignment and isolated medial JSN (at both thresholds) or isolated medial osteophytes (at the ≥1 threshold) in our study could partly reflect the imprecise nature of our measure of malalignment using intermalleolar and intercondylar distances compared to the full‐extremity radiograph measures of alignment used by Elahi et al 15. On the other hand, their finding of no association between compartmental PF joint JSN and BMI could be due to a lack of power, given the small sample size in their study (n = 86). Clearly, more studies are needed to elucidate the mechanisms underlying the development of medial and lateral PF joint OA.

Stefanik et al 20 showed that, despite a high prevalence of MRI‐detected cartilage damage in the medial PF joint compartment, knee pain was more common and more severe when OA was either isolated to or inclusive of the lateral PF joint compartment. In contrast, we found the highest pain scores in knees with mixed compartmental PF joint OA, and when compared with the neither compartmental PF joint OA category, the difference in median pain score was highest in knees with mixed compartmental PF joint OA. In the current study, knees with isolated lateral PF joint OA, whether defined by JSN or osteophytes, had higher median pain scores than knees with isolated medial PF joint OA, but these differences were mostly modest (<0.5 point on a 0–10 NRS) and clinically nonsignificant. A possible explanation for these findings is a lack of power in the current study to detect clinically meaningful differences in pain scores between isolated lateral and isolated medial PF joint OA. However, if our findings are correct, they suggest a need to rethink current treatment strategies for PF joint OA that attempt to realign the patella medially.

Furthermore, our results suggest that the relationship between compartmental radiographic PF joint OA and clinical outcomes may vary depending on the morphologic feature and severity threshold used to define PF joint OA. These findings require confirmation in other radiograph‐based symptomatic PF joint OA cohorts. If confirmed, we suspect that this could be one reason for the conflicting results of trials of conservative treatments for PF joint OA to date. Trials have often recruited participants on the basis of definite osteophytes 5, 6, 7 or moderate JSN (JSN ≥2) in the PF joint 6, mostly excluding participants with probable narrowing in the PF joint (JSN ≥1). The results of the current study suggest that inclusion of symptomatic patients with probable PF joint narrowing might lead to better patient selection for clinical trials.

There are a number of limitations to our study. We did not collect data on patellar malalignment, which is an important risk factor for PF joint OA 21. However, studies have found no association between patellar malalignment and clinical symptoms 21, 40, and the absence of these data in our cohort is not likely to bias our conclusions about the clinical significance of compartmental PF joint OA. Additionally, given the cross‐sectional design of the current study, we are unable to comment on the temporality of the relationship between compartmental PF joint OA and the clinical outcomes of pain and functional limitation.

In conclusion, our cross‐sectional study using radiography in community‐dwelling symptomatic adults suggests that isolated lateral PF joint OA is more common than isolated medial PF joint OA, and it is more consistently associated with known OA risk factors. Isolated lateral PF joint OA is associated with higher pain and function scores than isolated medial PF joint OA, particularly when defined using JSN rather than osteophytes. These differences, however, were generally modest and clinically nonsignificant in the present study. Future longitudinal research should investigate the relative incidence and progression of lateral and medial PF joint compartmental disease and their determinants.

## AUTHOR CONTRIBUTIONS

All authors were involved in drafting the article or revising it critically for important intellectual content, and all authors approved the final version to be submitted for publication. Dr. Peat had full access to all of the data in the study and takes responsibility for the integrity of the data and the accuracy of the data analysis.

### Study conception and design

Ukachukwu, Peat.

### Acquisition of data

Duncan, Peat.

### Analysis and interpretation of data

Ukachukwu, Belcher, Marshall, Stefanik, Crossley, Thomas, Peat.

## Supporting information

Supplementary Data Table S1. Summary of studies reporting relative frequency of medial vs. lateral PFJOA

## References

[1] McAlindon T , Snow S , Cooper C , Dieppe P. Radiographic patterns of osteoarthritis of the knee joint in the community: the importance of the patellofemoral joint. Ann Rheum Dis 1992;51:844–9. 1632657 10.1136/ard.51.7.844 PMC1004766

[2] Hinman RS , Crossley KM. Patellofemoral joint osteoarthritis: an important subgroup of knee osteoarthritis. Rheumatology (Oxford) 2007;46:1057–62. 17500072 10.1093/rheumatology/kem114

[3] Duncan R , Peat G , Thomas E , Wood L , Hay E , Croft P. Does isolated patellofemoral osteoarthritis matter? Osteoarthritis Cartilage 2009;17:1151–5. 19401244 10.1016/j.joca.2009.03.016

[4] Duncan R , Peat G , Thomas E , Hay EM , Croft P. Incidence, progression and sequence of development of radiographic knee osteoarthritis in a symptomatic population. Ann Rheum Dis 2011;70:1944–8. 21810840 10.1136/ard.2011.151050

[5] Quilty B , Tucker M , Campbell R , Dieppe P. Physiotherapy, including quadriceps exercises and patellar taping, for knee osteoarthritis with predominant patello‐femoral joint involvement: randomized controlled trial. J Rheumatol 2003;30:1311–7. 12784408

[6] Hunter DJ , Harvey W , Gross KD , Felson D , McCree P , Li L , et al. A randomized trial of patellofemoral bracing for treatment of patellofemoral osteoarthritis. Osteoarthritis Cartilage 2011;19:792–800. 21232620 10.1016/j.joca.2010.12.010 PMC3090698

[7] Crossley KM , Vicenzino B , Schache AG , Pandy MG , Hinman RS. Targeted physiotherapy treatment for patellofemoral osteoarthritis: a randomised clinical trial. Osteoarthritis Cartilage 2014;Suppl 22:S431. 10.1016/j.joca.2015.04.024 25960116

[8] Callaghan MJ , Parkes MJ , Hutchinson CE , Gait AD , Forsythe LM , Marjanovic EJ , et al. A randomised trial of a brace for patellofemoral osteoarthritis targeting knee pain and bone marrow lesions. Ann Rheum Dis 2015;74:1164–70. 25596158 10.1136/annrheumdis-2014-206376 PMC4771926

[9] Ficat RP , Hungerford DS. Disorders of the patella‐femoral joint. Baltimore: The Williams and Wilkins Co; 1977.

[10] Hehne HJ. Biomechanics of the patellofemoral joint and its clinical relevance. Clin Orthop Relat Res 1990;258:73–85. 2394060

[11] Elias JJ , Wilson DR , Adamson R , Cosgarea AJ. Evaluation of a computational model used to predict patellofemoral contact pressure distribution. J Biomech 2004;37:295–302. 14757448 10.1016/s0021-9290(03)00306-3

[12] Goudakos IG , König C , Schöttle PB , Taylor WR , Singh NB , Roberts I , et al. Stair climbing results in more challenging patellofemoral contact mechanics and kinematics than walking at early knee flexion under physiological‐like quadriceps loading. J Biomech 2009;42:2590–6. 19656517 10.1016/j.jbiomech.2009.07.007

[13] Akbarshahi M , Fernandez JW , Schache AG , Pandy MG. Subject‐specific evaluation of patellofemoral joint biomechanics during functional activity. Med Eng Phys 2014;36:1122–33. 24998901 10.1016/j.medengphy.2014.06.009

[14] Iwano T , Kurosawa H , Tokuyama H , Hoshikawa Y. Roentgenographic and clinical findings of patellofemoral osteoarthrosis with special reference to its relationship to femorotibial osteoarthrosis and etiologic factors. Clin Orthop Relat Res 1990;258:190–7. 2302884

[15] Elahi S , Cahue S , Felson D , Engelman L , Sharma L. The association between varus–valgus alignment and patellofemoral osteoarthritis. Arthritis Rheum 2000;43:1874–80. 10943879 10.1002/1529-0131(200008)43:8<1874::AID-ANR25>3.0.CO;2-2

[16] Gross KD , Niu J , Stefanik JJ , Guermazi A , Roemer FW , Sharma L , et al. Breaking the law of valgus: the surprising and unexplained prevalence of medial patellofemoral cartilage damage. Ann Rheum Dis 2012;71:1827–32. 22534825 10.1136/annrheumdis-2011-200606 PMC4011177

[17] Ratzlaff C , Duryea JW. More evidence for breaking the “law of valgus”: imaging evidence for higher prevalence and volume of bone marrow lesions in the medial patellofemoral joint. Arthritis Rheum 2013;65:S482.

[18] Ratzlaff C , Russell R , Duryea J. Quantitatively‐measured bone marrow lesions in the patellofemoral joint: distribution and association with pain. Osteoarthritis Cartilage 2014;22:S247–8.

[19] Hayashi D , Felson DT , Niu J , Hunter DJ , Roemer FW , Aliabadi P , et al. Pre‐radiographic osteoarthritic changes are highly prevalent in the medial patella and medial posterior femur in older persons: Framingham OA study. Osteoarthritis Cartilage 2014;22:76–83. 24185108 10.1016/j.joca.2013.10.007 PMC3947221

[20] Stefanik JJ , Gross KD , Guermazi A , Felson DT , Roemer FW , Zhang Y , et al. The relation of MRI‐detected structural damage in the medial and lateral patellofemoral joint to knee pain: the multicenter and Framingham osteoarthritis studies. Osteoarthritis Cartilage 2015;23:565–70. 10.1016/j.joca.2014.12.023 PMC4368472 25575967

[21] Hunter D , Zhang Y , Niu J , Felson D , Kwoh K , Newman A , et al. Patella malalignment, pain and patellofemoral progression: The Health ABC study. Osteoarthritis Cartilage 2007;15:1120–7. 17502158 10.1016/j.joca.2007.03.020 PMC2042530

[22] Kalichman L , Zhang Y , Niu J , Goggins J , Gale D , Felson DT , et al. The association between patellar alignment and patellofemoral joint osteoarthritis features: an MRI study. Rheumatology (Oxford) 2007;46:1303–8. 17525117 10.1093/rheumatology/kem095

[23] Stefanik JJ , Zhu Y , Zumwalt AC , Gross KD , Clancy M , Lynch JA , et al. Association between patella alta and the prevalence and worsening of structural features of patellofemoral joint osteoarthritis: the Multicenter Osteoarthritis Study. Arthritis Care Res (Hoboken) 2010;62:1258–65. 20506169 10.1002/acr.20214 PMC2943040

[24] Cahue S , Dunlop D , Hayes K , Song J , Torres L , Sharma L. Varus–valgus alignment in the progression of patellofemoral osteoarthritis. Arthritis Rheum 2004;50:2184–90. 15248216 10.1002/art.20348

[25] Crossley KM , Lentzos J , Vicenzino B , Hinman RS. Prevalence of radiographic patellofemoral and tibiofemoral osteoarthritis in individuals with chronic anterior knee pain: data from a randomised clinical trial. Osteoarthritis Cartilage 2012;20:S266–7.

[26] Peat G , Thomas E , Handy J , Wood L , Dziedzic K , Myers H , et al. The Knee Clinical Assessment Study CAS(K): a prospective study of knee pain and knee osteoarthritis in the general population. BMC Musculoskelet Disord 2004;5:1–9. 15028109 10.1186/1471-2474-5-4 PMC368438

[27] Peat G , Thomas E , Handy J , Wood L , Dziedzic K , Myers H , et al. The Knee Clinical Assessment Study CAS(K): a prospective study of knee pain and knee osteoarthritis in the general population. Baseline recruitment and retention at 18 months. BMC Musculoskelet Disord 2006;7:1–11. 16542454 10.1186/1471-2474-7-30 PMC1435895

[28] Buckland‐Wright C. Which radiographic techniques should we use for research and clinical practice? Best Pract Res Clin Rheumatol 2006;20:39–55. 16483906 10.1016/j.berh.2005.08.002

[29] Altman RD , Hochberg M , Murphy A , Wolfe F , Lesquesne M. Atlas of individual radiographic features in osteoarthritis. Osteoarthritis Cartilage 1995;3:3–70. 8581752

[30] Duncan RC , Hay EM , Saklatvala J , Croft PR. Prevalence of radiographic osteoarthritis: it all depends on your point of view. Rheumatology (Oxford) 2006;45:757–60. 16418199 10.1093/rheumatology/kei270

[31] Duncan R , Peat G , Thomas E , Wood L , Hay E , Croft P. How do pain and function vary with compartmental distribution and severity of radiographic knee osteoarthritis? Rheumatology (Oxford) 2008;47:1704–7. 18805874 10.1093/rheumatology/ken339

[32] Von Korff M , Ormel J , Keefe FJ , Dworkin SF. Grading the severity of chronic pain. Pain 1992;50:133–49. 1408309 10.1016/0304-3959(92)90154-4

[33] Bellamy N. WOMAC Osteoarthritis Index: a user's guide. London, Ontario: University of Western Ontario; 1996.

[34] World Health Organization . The burden of musculoskeletal conditions at the turn of the millennium. Geneva: World Health Organization; 2003.

[35] Felson DT , Naimark A , Anderson J , Kazis L , Castelli W , Meenan RF. The prevalence of knee osteoarthritis in the elderly: the Framingham osteoarthritis study. Arthritis Rheum 1987;30:914–8. 3632732 10.1002/art.1780300811

[36] Peat G , Thomas E , Duncan R , Wood L , Wilkie R , Hill J , et al. Estimating the probability of radiographic osteoarthritis in the older patient with knee pain. Arthritis Rheum 2007;57:794–802. 17530679 10.1002/art.22785

[37] Lacey RJ , Thomas E , Duncan RC , Peat G. Gender difference in symptomatic radiographic knee osteoarthritis in the knee clinical assessment CAS(K): a prospective study in the general population. BMC Musculoskelet Disord 2008;9:1–8. 18547403 10.1186/1471-2474-9-82 PMC2443794

[38] Goodfellow J , Hungerford D , Zindel M. Patello‐femoral joint mechanics and pathology. 1. Functional anatomy of the patello‐femoral joint. J Bone Joint Surg Br 1976;58:287–90. 956243 10.1302/0301-620X.58B3.956243

[39] Goodfellow J , Hungerford D , Woods C. Patello‐femoral joint mechanics and pathology. 2. Chondromalacia patellae. J Bone Joint Surg Br 1976;58:291–9. 956244 10.1302/0301-620X.58B3.956244

[40] Iijima H , Fukutani N , Aoyama T , Fukumoto T , Uritani D , Kaneda E , et al. Clinical impact of coexisting patellofemoral osteoarthritis in Japanese patients with medial knee osteoarthritis. Arthritis Care Res (Hoboken) 2016;68:493–501. 26315986 10.1002/acr.22691

