# Supplementary material for: Clinical Significance of Medial Versus Lateral Compartment Patellofemoral Osteoarthritis: Cross‐Sectional Analyses in an Adult Population With Knee Pain
Source: Arthritis Care Res (Hoboken). 2017 Jun 27;69(7):943–51. doi: 10.1002/acr.23110 (PMC5519922; doi:10.1002/acr.23110)
Supplement: Supplementary file 1 — Supplementary Data Table S1. Summary of studies reporting relative frequency of medial vs. lateral PFJOA [file ACR-69-943-s001.doc]

**Supplementary Data Table S1. Summary of studies reporting relative frequency of medial vs. lateral PFJOA**

|  | **Gross et al 2012** | **Ratzlaff et al 2014** | **Hayashi et al 2014** | **Iwano et al 1990** | **Elahi et al 2000** | **Cahue et al 2004** | **Crossley et al 2012** |
| --- | --- | --- | --- | --- | --- | --- | --- |
| Study type | Cross sectional | Cross sectional | Cross sectional | Cross sectional | Cross sectional | Longitudinal | Cross sectional |
| Imaging modality | MRI | MRI | MRI | X-ray, skyline view with knee flexion of 45° | X-ray, skyline view with knee flexion of 30° | X-ray, skyline view with knee flexion of 30° | X-ray, skyline view with knee flexion between 30 - 40° |
| OA Definition | WORMS - Cartilage morphology only: Applied four different cut-offs ≥2, ≥3, ≥4 and ≥5 | Automated BML volume: assessed continuously | WORMS OP Grade ≥2 and Cartilage morphology: (Grade ≥2 reported separately | Knees with ALL three features – osteophytes, JSN and subchondral sclerosis, PLUS JSN < 3mm | OARSI atlas – asymmetric PF JSN and presence of PF OP | OARSI atlas - PF JSN: an increase grade ≥1 indicated progression | Kellgren Lawrence grade: Described frequencies in mild and moderate/severe groups. |
| Population/Participants description | BOKS – adult symptomatic knee OA cohort  FOA – adult population-based cohort, no selection based on the presence/ absence of knee OA  MOST – adults with existing knee OA or at high risk of developing the disease | Osteoarthritis Initiative Progression Cohort – Adults with definite knee OA | Framingham Community Cohort – radiographically ‘normal’ knees i.e. no ROA; though used only lateral x-ray views and no skyline views | Adults with definite knee OA (Japanese population) – knees with moderate to severe OA | Adults with definite symptomatic knee OA | Adults with definite symptomatic knee OA | Baseline RCT sample – individuals with chronic anterior knee pain |
| Mean age (SD), years | BOKS 66.7(9.4);  FOA 65(8.9)  MOST 62.1(8.0) | - | 62.3(8.4) | 62.6 | 66 (11) | 68.4(10.8) | 54 (10) |
| Sex (Female) | BOKS 41.1%  FOA 56%  MOST 61% | 48% | 55.2% | 92.4% | 71% | 71% | 51% |
| BMI (SD), kg/m^2^ | BOKS 30.7(4.8)  FOA 29.2(5.5)  BMI 30(4.9) | - | 27.9(5.1) | - | 31(7) | 30.6(6.1) | 27(4) |
| Findings | Medial PFJOA more prevalent than lateral, except at higher OA definition cut-offs where lateral PFJOA prevalence approximated (and slightly exceeded) that of medial PFJOA. Finding consistent across all three study populations. | Prevalence: medial – 46%, lateral – 44%.  PFJ BML at least as common medially as laterally | Prevalence of pre-radiographic OA changes - Cartilage morphology: medial 47.7%, lateral 29.9%  Osteophytes: medial 24.6%, lateral 34% | Lateral PFJOA was more common than medial PFJOA | Lateral PFJOA was more common than medial PFJOA; knees with PFJOA were more often valgus than knees with isolated TFJOA | Lateral PFJOA progression was more common than medial PFJOA progression | Prevalence of medial and lateral PFJOA appear equal |

WORMS – whole organ magnetic resonance imaging score, BML – bone marrow lesion, JSN – joint space narrowing, OP – osteophytes, PFJOA – patellofemoral joint osteoarthritis, PF patellofemoral
